# Management of uterine rupture: a case report and review of the literature

**DOI:** 10.1186/s13104-016-2295-9

**Published:** 2016-11-21

**Authors:** Thomas Obinchemti Egbe, Gregory Edie Halle-Ekane, Charlotte Nguefack Tchente, Jacques Ernest Nyemb, Eugene Belley-Priso

**Affiliations:** 1 Department of Obstetrics and Gynecology, Douala General Hospital, Douala, Cameroon; 2 Faculty of Health Sciences, University of Buea, Buea, Cameroon; 3 Faculty of Medicine and Pharmaceutical Sciences, University of Douala, Douala, Cameroon; 4 Operating Theatre, Douala General Hospital, Douala, Cameroon; 5 Faculty of Medicine and Biomedical Sciences, University of Yaounde 1, Yaoundé, Cameroon

**Keywords:** Uterine rupture, Emergency, Laparotomy, Hysterectomy, Transfusion, Blood products

## Abstract

**Background:**

Maternal morbidity and mortality has been a major World Health Organization concern over the years, especially in sub-Saharan Africa. This paper reports uterine rupture with severe hypovolemic shock managed at the Douala General Hospital, Cameroon. Early clinical diagnosis is paramount to maternal survival.

**Case presentation:**

Mrs. MM aged 25 years, G3P2012, of the Bamileke tribe in Cameroon was admitted to our Department in hypovolemic shock BP = 70/40 mmHg, pulse 120 beats per minute, with altered consciousness (Glasgow Coma Scale = 13). She has a history of missed abortion at 19 weeks gestation and an attempt to evacuate the uterus with misoprostol that led to uterine rupture. She underwent a total abdominal hysterectomy and blood transfusion. Her post-operative stay in hospital was uneventful.

**Conclusion:**

Uterine rupture is a complication that can be eliminated under conditions of best obstetric practice. To attain this objective, use of misoprostol in primary health facilities should be stopped or proper management of the medication instituted. The survival of patients after uterine rupture depends on the time interval between rupture and intervention, and the availability of blood products for transfusion.

## Background

Maternal mortality, one of the major concerns of the World Health Organization, remains high in most of sub-Saharan Africa [1, 2]. In the particular case of Mezam Division, Cameroon, the leading causes are Postpartum Hemorrhage (30.43%), unsafe abortion (26.09%), and pregnancy-induced hypertension (14.49%) [1, 2].

Misoprostol is a prostaglandin E1 analog that was originally used for the prevention and treatment of peptic ulcer disease [3]. It has been recently used in the treatment of post-partum hemorrhage and complications of abortion [4]. The optimal dose for this medication has been a controversial issue and practitioners, especially those with little or no experience with the manipulation of this medication, sometimes have difficulties that could lead to life-threatening consequences like uterine rupture [5].

We are reporting a case of uterine rupture for second trimester evacuation of a dead fetus that was managed at the department of Obstetrics and Gynecology, Douala General Hospital, Cameroon.

## Case presentation

Mrs. MM aged 25 years, G3P2012, of the Bamileke tribe in Cameroon was admitted to our Department in hypovolemic shock BP = 70/40 mmHg, pulse 120 beats per minute with altered consciousness (Glasgow Coma Score = 13). She has a history of missed abortion at 19 weeks gestation diagnosed by ultrasonography 3 days prior to admission at our Department, and an attempt to evacuate the uterus with an unknown dose of misoprostol before she went into shock. At the time of the uterine evacuation, the pregnancy was 24 weeks 2 days gestation calculated from her last menstrual period. On examination, the conjunctivae were pale and the pulse rate 120 beats per minute. The abdomen was distended and tender on palpation. There was a fluid thrill, shifting dullness and mild vaginal bleeding. An emergency positive culdocentesis was done.

During this period, the anesthesiologist had been called who secured an intravenous line with a 14 G catheter, obtained blood for Full blood count, coagulation studies, typing and cross match. 1500 mL of blood was secured from the laboratory and she underwent an emergency laparotomy with a sub-umbilical mid-line incision.

**Fig. 1:**
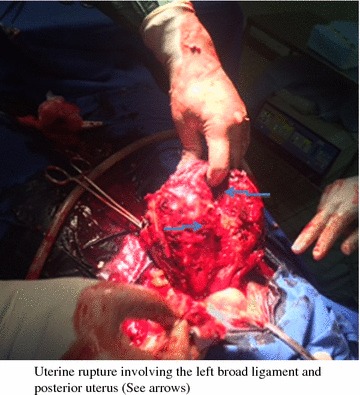
Posterior uterine rupture extending from the fundus to the isthmus and affecting the left broad ligament and uterine vessels (See *arrows*)

**Fig. 2:**
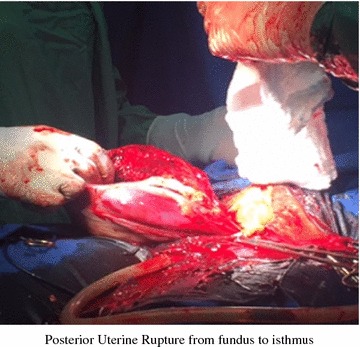
Posterior wall uterine rupture extending from the fundus to the isthmus

During surgery, we found that there was hemoperitoneum estimated at about 2500 mL and the uterus was completely torn posteriorly from the fundus to the isthmus and extending to the left broad ligament with involvement of the ascending branch of the uterine artery (Figs. 1, 2). The fetus was found in the peritoneal cavity completely macerated. We carried out a total abdominal hysterectomy and peritoneal toileting. The patient was transfused 1500 mL of whole blood during surgery. She was then transferred to the intensive care unit (ICU) where she was followed up for 48 h. Her hemoglobin level the day after surgery was 6.4 g/dL. She received a further 1000 mL of packed cells at the ICU, making a total of 2500 mL blood received by the patient. She remained at the Department of Obstetrics and Gynecology for 5 more days and her hemoglobin level on discharge was 8.1 g/dL. Patient was discharged on hematinics and vitamins.

## Conclusions

Maternal mortality has been one of the millennium development goals (MDG 5) and although progress has gradually been made in reducing maternal mortality, action is needed to meet the sustainable development goal (SDG) 2030 target [6]. Uterine rupture and maternal death from hemorrhage is a preventable complication of childbirth in sub-Saharan Africa.

## Prevalence of uterine rupture

Uterine rupture is a serious obstetrical condition associated with maternal mortality. In a systematic review by Justus Hofmeyr et al., uterine rupture was reported to be lower in a community-based study (median 0.053%, range 0.016–0.030%) compared to facility-based study (0.031, 0.012–2.9%). This prevalence was also higher in less developed countries (sub-Saharan Africa especially) than in the developed countries [7]. Prual et al. in a case fatality study of maternal morbidity from 23 West African countries and 20326 pregnant women between 32 and 36 weeks amenorrhea during delivery and up to 60 days post partum reported that uterine rupture accounted for 0.12 per 100 live births [8]. Nguefack et al. in Douala reported that the prevalence of uterine rupture was 0.4% (1:249) [9]. Another study in Ethiopia reported a prevalence of 0.9% [10].

## Vaginal birth after cesarean

There is a steady decrease in the rate of vaginal birth after cesarean (VBAC) [11]. In a study in Ghana, a fetal weight greater than 3.45 kg tripled the likelihood of having a repeat cesarean delivery (CD), and the probability of having a repeat CD was 50% for a fetal weight of 3.70 kg [12, 13]. This fact should be considered when counseling women for VBAC [13].

The increasing number of women with a previous cesarean section and the decreasing rates of VBAC seem to suggest that cesarean sections are gaining more currency. One of the greatest concerns regarding VBAC is the potential for uterine rupture. Studies have reported that the incidence rate of uterine rupture in women who attempt VBAC was 9.8 per 1000 and prior vaginal delivery was associated with a lower risk of uterine rupture (adjusted odds ratio [OR] 0.40, 95% CI 0.20–0.81) [14].

## Use of oxytocin and prostaglandins

With the advent of misoprostol, a prostaglandin E1 analog is cheap and accessible to most health facilities in Cameroon and most countries in sub-Saharan Africa. As a result, the rates of uterine rupture have increased noticeably. The study by Nguefack et al. reported that 71% of cases with uterine rupture used misoprostol [9]. Studies in Bangladesh and India report the use of oxytocin by unqualified allopathic practitioners (UAP) providing health services to the poor [15]. These providers are close to homes, willing to make house calls, trusted by the community, have longer working hours and offer services at lower costs. UAPs, comprising village doctors (VDs) and unlicensed drug sellers, have limited training of a few weeks to a few months from semiformal private institutions, focused on common illnesses and diseases, and rarely on labour or delivery. These training institutions are unregulated and do not follow a standard [15]. This type of practice should be discouraged because it is associated with obstetric and neonatal complications such as uterine rupture [15].

## Partogram use

Health personnel are almost forgetting the good practice of using the partogram for labor follow-up. Egbe et al. in the Bamenda Health District, Cameroon, showed that 58.2% deliveries were followed up with the partogram, only 1% of which were filled to standard [16]. Wacker et al. in Burkina Faso reported 46.6% partogram use [17] while Ogwang et al. in Rukungiri District in Uganda reported 30% use [18]. These low rates of partogram use could have obstetric consequences, especially given the high likelihood that, under such circumstances, parturients are administered oxytocin or prostaglandins and are not properly followed up by hourly or two-hourly examinations.

## Cervical ripening with a trans-cervical foley

In a study of patients with a prior cesarean being induced with the trans-cervical foley bulb, the rate of uterine rupture was 1.1% with spontaneous labor, 1.2% with induction with amniotomy, and 1.6% with use of a trans-cervical Foley bulb. Therefore, labor induction using a trans-cervical Foley catheter was not associated with an increased risk of uterine rupture [19]. This may have been the appropriate method for our patient although she was at no particular risk of uterine rupture.

## Inter-pregnancy interval

Several studies have shown that the shorter the time between a cesarean delivery and a subsequent delivery, the higher the rate of uterine rupture. Commonly, thresholds of 18 and 24 months have been examined. Adjusted odds ratios range from 2.5 to 3 for an increased rate of uterine rupture in the women with less time between deliveries. The biologic plausibility of this effect is related to the amount of time required for the uterine scar to heal completely and to nutritional Factors [20–22].

## Hysterotomy technique

Women with a classical incision that run vertically on the corpus uteri run a higher risk of uterine rupture than those with a low uterine segment transverse incision [23]. Patients with a classical hysterotomy are likely to rupture during pregnancy and studies have shown that they should be delivered by 36–37 weeks gestation. However, others suggest a planned cesarean delivery at 38 weeks gestation [24]. Furthermore, a single-layer closure of the previous lower segment incision is the most influential factor and is associated with a fourfold increase in the risk of uterine rupture compared with a double-layer closure [25].

## Uterine rupture in unscarred uterus

In a study of 32 080 deliveries in JIPMER (India), 93 (0.28%) women had a ruptured uterus. The majority (77%) had a scarred uterus. Among women with unscarred uterus, 14 presented with rupture and seven of these women were induced in hospital. It was then concluded that the strongest association of ruptured uterus was with previous scarred uterus, multiparity and <18 months’ duration from the last cesarean section [26]. In another population-based study in the Netherlands, the incidence of uterine rupture was comparable with other Western countries. Although much attention is paid to scar rupture associated with uterotonic agents, 13% of ruptures occurred in unscarred uteri and 72% occurred during spontaneous labour [27].

Reports from the study in Mali show that uterine rupture occurred in 87.4% (415/475) of cases in an unscarred uterus vs 12.6% (60/475) in a scarred uterus. Observed risk factors for primary uterine rupture included: contracted pelvis, 12.0% (57/475); fetal macrosomia 9.7% (46/475); contracted pelvis associated with macrosomia 3.4% (16/475). Malpresentation was recorded in 12.4% (59/475). Dystocia associated with oxytocin and/or traditional medicines labor augmentation has been observed in 12.6% of cases (60/475). Grand multiparity (≥7 deliveries in obstetric history) accounted for 12.4% (59/475) of all uterine ruptures while short inter-pregnancy interval has been observed in 12.0% of all uterine ruptures (57/475) [28].

Our patient was not at particular risk for uterine rupture. She was administered misoprostol to effect uterine evacuation of a dead fetus in a primary care centre with no facilities or skilled personnel to carry out a cesarean section. The patient would have lost her life had intervention not been prompt. She owed her life to her timely action. She came to our Department relatively early, about 30 min after the incident, and we intervened immediately, aided by the fact that compatible blood was available in the blood bank.

Uterine rupture is a clinical diagnosis and there must be a high index of suspicion by the healthcare provider. Risk factors for such ruptures may include previous uterine scar, short birth spacing, and use of uterotonic (oxytocin/prostaglandin) medications [9, 29, 30]. A scarred uterus is not a necessary pre-condition for uterine rupture. Turner et al. reported that uterine rupture in the majority of cases is associated with poor and traumatic obstetric practice [31]. Our patient was administered an unknown dosage of misoprostol that resulted in the rupture.

This case stresses the importance of good obstetric practice and the need for qualified medical and paramedical staff. It cautions that medications like oxytocin and prostaglandins should be manipulated under specialized care and, finally, that health establishments should not handle deliveries unless they are equipped for the complications of labour and delivery.

Finally, uterine rupture is a complication that can be eliminated if best obstetric practice is ensured. The survival of patients after uterine rupture depends on the time interval between rupture and intervention and the availability of blood products for transfusion. CARE guidelines/methodology were adhered to in the preparation of this manuscript.

## Abbreviations

GCS: glasgow coma score
ICU: intensive care unit
CS: cesarean section
CD: cesarean delivery
MDG: millenium development goals
SDG: sustainable development goals
VBAC: vaginal birth after cesarean
UAP: unqualified allopathic practitioners
VD: village doctor
